# A novel *PRKAG2* mutation in a Chinese family with cardiac hypertrophy and ventricular pre-excitation

**DOI:** 10.1038/s41598-017-02455-z

**Published:** 2017-05-25

**Authors:** Kun-Qi Yang, Chao-Xia Lu, Ying Zhang, Yan-Kun Yang, Jia-Cheng Li, Tian Lan, Xu Meng, Peng Fan, Tao Tian, Lin-Ping Wang, Ya-Xin Liu, Xue Zhang, Xian-Liang Zhou

**Affiliations:** 1 Department of Cardiology, Fuwai Hospital, National Center for Cardiovascular Diseases, Chinese Academy of Medical Sciences and Peking Union Medical College, Beijing, China; 2 McKusick-Zhang Center for Genetic Medicine, State Key Laboratory of Medical Molecular Biology, Institute of Basic Medical Sciences, Chinese Academy of Medical Sciences and Peking Union Medical College, Beijing, China; 3 Department of Magnetic Resonance Imaging, Cardiovascular Imaging and Intervention Center, Fuwai Hospital, National Center for Cardiovascular Diseases, Chinese Academy of Medical Sciences and Peking Union Medical College, Beijing, China

## Abstract

PRKAG2 syndrome is a rare autosomal dominant inherited disorder that is characterized by cardiac hypertrophy, ventricular pre-excitation and conduction system abnormalities. There is little knowledge in cardiovascular magnetic resonance (CMR) characteristics of PRKAG2 cardiomyopathy. This study investigated the genetic defect in a three-generation Chinese family with cardiac hypertrophy and ventricular pre-excitation using whole-exome sequencing. A novel missense mutation, c.1006 G > T (p.V336L), was identified in *PRKAG2*. This mutation had not been identified in the ExAC database, and the prediction result of MutationTaster indicated a deleterious effect. Furthermore, it cosegregated with the disease in the present family and was absent in unrelated 300 healthy controls. cDNA analysis did not detect any splicing defects, although the variant occurred in the first base of exon 9. CMR evaluation in five affected members showed diffuse hypertrophy in a concentric pattern, with markedly increased left ventricular mass above age and gender limits (median 151.3 g/m^2^, range 108.4–233.4 g/m^2^). Two patients in progressive stage and one patient with sudden cardiac death exhibited extensive subendocardial late gadolinium enhancement. In conclusion, molecular screening for *PRKAG2* mutations should be considered in patients who exhibit cardiac hypertrophy coexisting with ventricular pre-excitation. CMR offers promising advantages for evaluation of PRKAG2 cardiomyopathy.

## Introduction

PRKAG2 syndrome (PS) is a rare, early-onset autosomal dominant inherited disorder, and is characterized by cardiac hypertrophy, ventricular pre-excitation (VPE) and progressive conduction abnormalities^1–3^. Little is known about the prevalence of this disorder. Murphy RT *et al*.^3^ estimated it to be 1% in patients with both hypertrophy cardiomyopathy (HCM) and premature sinoatrial or atrioventricular conduction disease. To date, the reported patients with genetically confirmed PS are less than 200^4^.

Since the first reports of *PRKAG2* mutations in 2001^1, 2, 5^, *PRKAG2* defects have been identified as the pathogenic molecular basis of PS. To date, 21 mutations associated with PS have been identified. The *PRKAG2* gene codes for the γ2 regulatory subunit of AMP-activated protein kinase (AMPK)^6^. AMPK is known as a fuel gauge that is ubiquitously expressed in eukaryotic cells, and which modulates cellular energy homeostasis by switching on ATP-generating pathways and switching off anabolic pathways in response to cellular stress^7^. The AMPK-γ2 subunit is most abundantly expressed in heart and is responsible for regulating AMPK activity by competitively binding either ATP or AMP^6, 8^.

Identification of *PRKAG2* defects provides a new insight into the molecular basis of unexplained left ventricular hypertrophy (LVH) beyond mutations in genes encoding the sarcomeric proteins. Distinct from sarcomeric HCM, the primary feature of myocardial histopathology in PRKAG2 cardiomyopathy is widespread intracellular vacuolation filled with glycogen, instead of myofiber disarray and interstitial fibrosis^3, 9–11^. Although cardiovascular magnetic resonance (CMR) has been widely used to evaluate myocardial morphology and function in sarcomeric HCM^12, 13^, and is increasingly used in the assessment of metabolic cardiomyopathies^14^, only a few cases with PS have been reported with CMR findings^11, 15–17^.

In this study, we investigated a three-generation Chinese family with cardiac hypertrophy and VPE. A novel *PRKAG2* heterozygous mutation was detected by whole-exome sequencing, and the diagnosis of PS was established in affected family members. We also conducted a phenotype analysis using CMR findings in five patients in the present family, and performed a review of the reported PS cases with CMR findings, to add more knowledge to the CMR characteristics in PRKAG2 cardiomyopathy.

## Results

### Clinical features

**Figure 1:**
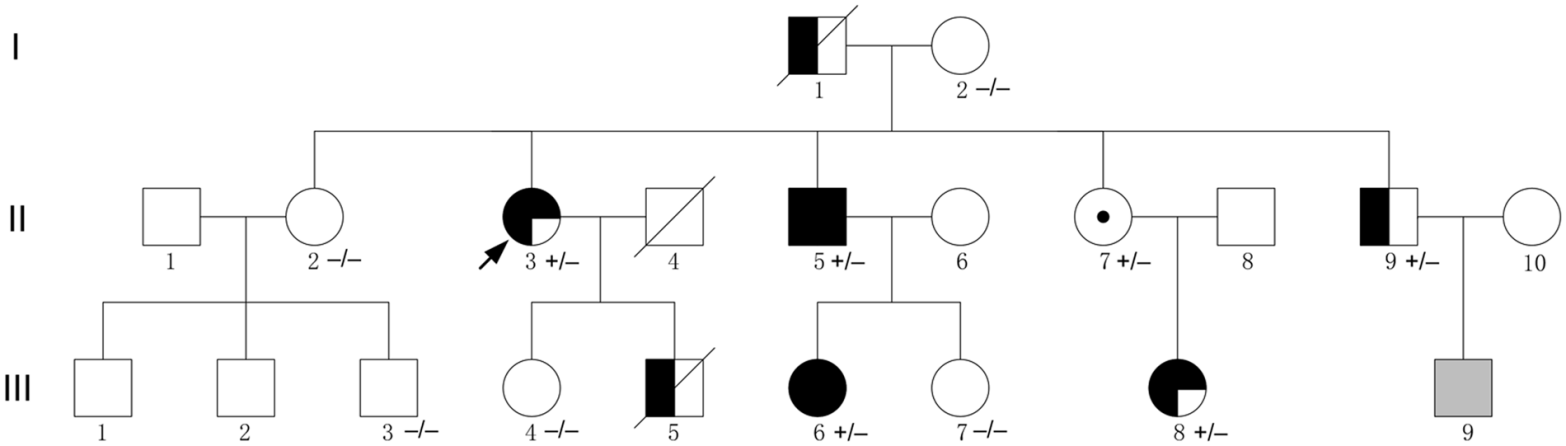
Pedigree of the family with PRKAG2 syndrome. Squares indicate males and circles indicate females. Filled symbols indicate the disease phenotype in affected individuals, i.e., cardiac hypertrophy (left half filled), ventricular pre-excitation (right-upper quadrant filled), or conduction system disease (right-lower quadrant filled). Open symbols represent unaffected individuals, symbols with dots represent mutation carriers without clinical manifestation, shading represents an uncertain clinical status and no sample was available. An arrow denotes the indexed subject and slants denote dead individuals. + indicates *PRKAG2* p.V336L mutation positive, and − indicates *PRKAG2* p.V336L mutation negative.

**Figure 2:**
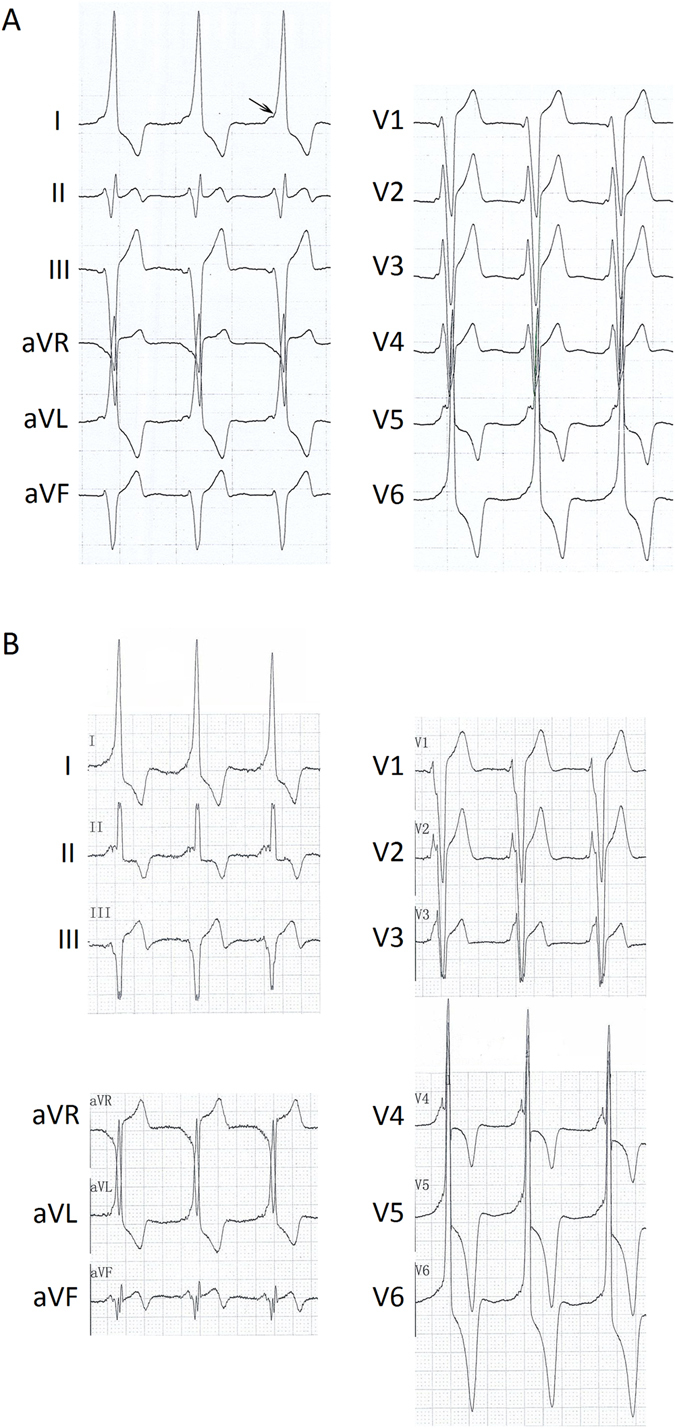
ECGs from the proband. (**A**) Resting ECG before ablation shows ventricular pre-excitation and delta waves (PR interval, 52 ms; QRS interval, 100 ms), with major QRS complex up waves of complexes in the V1 lead and major QRS complex down waves in V5–6 leads, indicative of a diagnosis of type B WPW syndrome. (**B**) Two years after ablation, short PR intervals (PR interval, 82 ms), enlarged ORS complexes (QRS interval, 190 ms), and deeply inverted T waves were detected in V5–6 leads. The arrow points to the delta wave.

Figure 1 presents the pedigree of a three-generation Chinese family with cardiac hypertrophy and VPE, which was consistent with autosomal dominant inheritance. Totally, there are 7 clinically affected subjects in this family, including two deceased members (individuals I-1 and III-5). The proband (individual II-3), a 53-year-old female, was referred to our hospital for family screening because of her son’s unexplained cardiac hypertrophy and sudden cardiac death (SCD) a month earlier. Abnormal recordings on resting ECG had been observed since a physical examination at age 32. Two years ago, she presented with paroxysmal palpitation and type B WPW syndrome was suggested by her resting ECG (Fig. 2A). An electrophysiological study was performed and showed a right overt accessory pathway (7 o’clock on the tricuspid annulus) which was successfully ablated. Echocardiography showed high-normal thickness in the interventricular septum (IVS, 12 mm) and left ventricular posterior wall (LVPW, 11 mm). On admission, she presented with a short PR interval and widened ORS interval by ECG, suggestive of VPE (Fig. 2B). Echocardiography showed IVS and LVPW hypertrophy with a maximum thickness of 20 mm.

**Figure 3:**
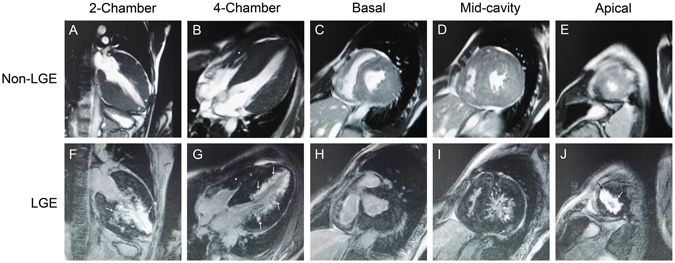
Normal and contrast-enhanced CMR images in individual III-5 who suffered from SCD at age 24. Two chamber and four chamber cine images show remarked LVH and mild RVH (▲), with apical involvement (*) (**A**,**B**). Basal, mid, and apical LV short-axis images show hypertrophy in almost all segments, with a maximal LV wall thickness of 33 mm and the maximal RV wall thickness of 14 mm (**C**–**E**). Arrows indicate late gadolinium enhancement. LV hypertrophic regions exhibit patchy, subendocardial and midmyocardial late gadolinium enhancement (**F**–**J**) (white and black arrows). SCD, sudden cardiac death; LVH, left ventricular hypertrophy; RVH, right ventricular hypertrophy; LV, left ventricle.

The proband’s son (individual III-5) died suddenly at age 24. He had a 7-year history of cardiac hypertrophy, with complaints of dizziness and amaurosis fugax after intense activity. Severe concentric LVH and mild right ventricular hypertrophy, with left ventricular outflow tract obstruction and impaired systolic function (left ventricular ejection fraction, LVEF, 46%), were demonstrated by CMR (Fig. 3). A 12-lead ECG performed two weeks before his death showed mild sinus bradycardia (heart rate, 56 bpm), high-voltage QRS complexes and abnormal ST-T interval changes. The proband’s father (individual I-1) died at 75 years old. He had a history of cardiac hypertrophy, atrial fibrillation and cerebral hemorrhage, and a permanent pacemaker was implanted at age 72.

Patient III-8, aged 24 years, developed hypertension eight years ago. Abnormalities in the kidneys, adrenal glands and renal arteries were excluded by ultrasound examinations. Echocardiography showed apical hypertrophy with a maximum thickness of 25 mm. The patient’s mother (individual II-7) had high-voltage QRS complexes in 12-lead ECG, but no cardiac hypertrophy or other abnormalities were detected by echocardiographic examination. Both patients II-5 and II-9, aged 49 and 45 years, respectively, had asymptomatic severe cardiac hypertrophy confirmed by CMR (see Supplementary Fig. S1). Patient III-6 had an 8-year history of asymptomatic cardiac hypertrophy, and had complained of fatigue after moderate activity over the past six months. The clinical details of the patients studied are shown in Table 1.

**Table 1:** Clinical characteristics of affected members in the current family with PRKAG2 syndrome.

Patient	Sex	Age, years	Age at onset, years	Symptoms	Echocardiography						ECG				Blood pressure, mm Hg	NYHA
					LVH	IVS, mm	LVPWT, mm	LVOTO	LVEF, %	E/A < 1	WPW syndrome	Pre-excitation	Conduction system disease	LVHV		
II-3	F	53	32	Dizziness, palpitation	+	14	9	−	68	+	+	+	−	+	140/80	I
II-5	M	49	38	Asymptomatic	+	22	20	−	70	−	–	+	+	+	130/80	I
II-9	M	45	45	Asymptomatic	+	31	13	−	75	+	−	−	−	−	136/82	I
III-5	M	24	16	Dizziness, amaurosis fugax (after intense activity)	+	22	30	−	53	+	−	−	−	+	120/74	II
III-6	F	27	18	Fatigue	+	29	21	−	55	+	−	+	+	+	120/70	II
III-8	F	24	24	Headache, dizziness	+	12	12	−	65	−	−	+	−	+	150/110	I

F, female; M, male; LVH, left ventricular hypertrophy; IVS, interventricular septum; LVPWT, left ventricular posterior wall thickness; LVOTO, left ventricular outflow tract obstruction; LVEF, left ventricular ejection fraction; WPW syndrome, Wolff-Parkinson-White syndrome; LVHV, Left ventricular high voltage.

### Identification of a novel *PRKAG2* missense mutation

**Figure 4:**
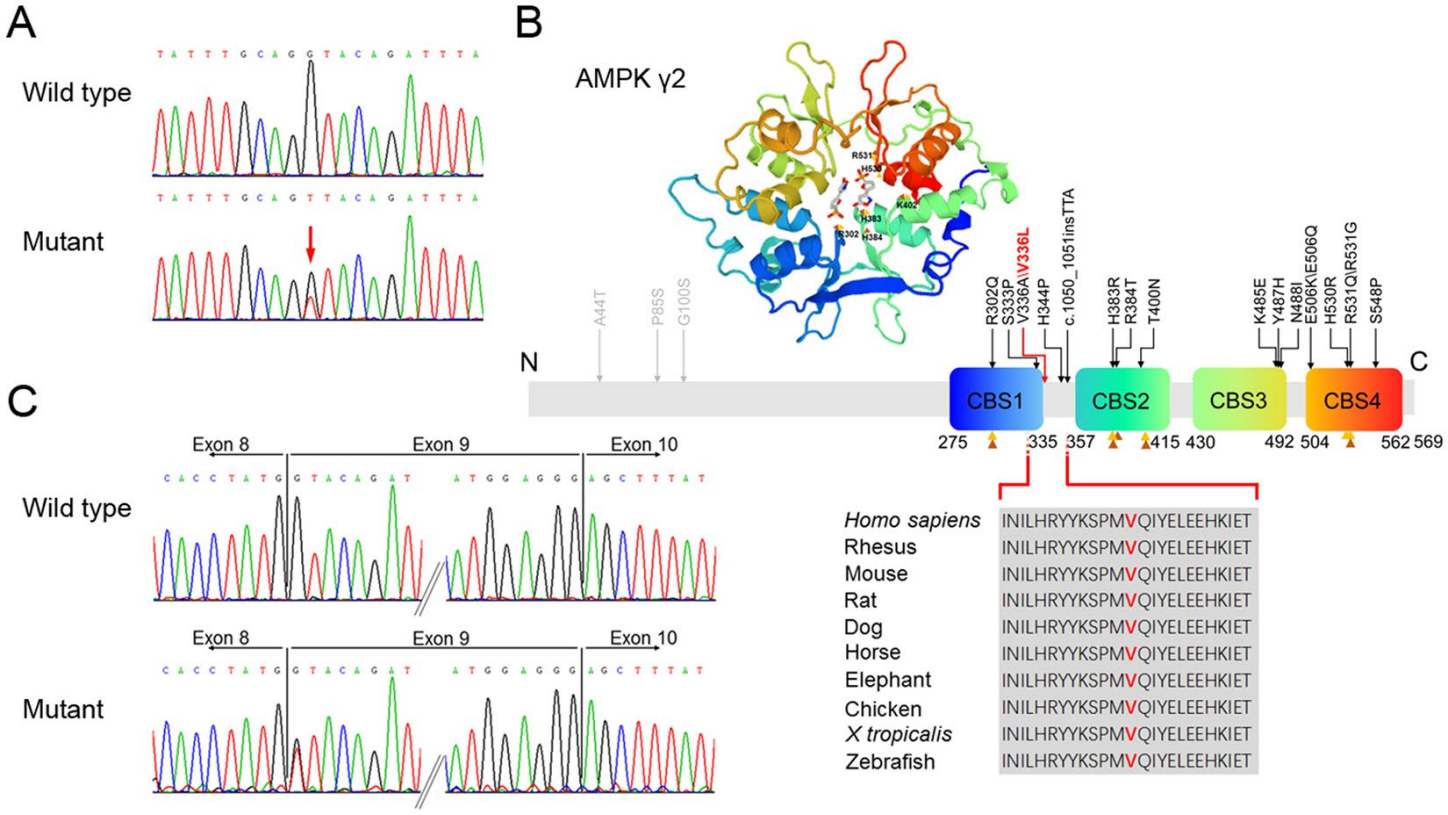
Genetic analysis identified a novel missense mutation V336L in a family with PRKAG2 syndrome. (**A**) DNA sequencing chromatogram shows a heterozygous G > T transition at nucleotide 1006 of *PRKAG2*. (**B**) Three-dimensional model and schematic of the AMPK-γ2 subunit and all identified *PRKAG2* mutations (mutations with grey symbols represent possible disease-causing mutation, mutations with black symbols represent disease-causing mutation; data obtained from Human Gene Mutation Database, www.hgmd.org). Residue V336 is located adjacent to the CBS1 domain and is highly conserved across species. Orange triangle symbols point to AMP binding sites and brown ones point to ATP binding sites (data obtained from UniProtKB, www.uniprot.org). (**C**) *PRKAG2* cDNA sequencing of the proband shows that the mutation does not cause exon skipping.

Whole-exome sequencing identified a heterozygous single-base alteration at position 1006 (c.1006 G > T) in *PRKAG2* (Fig. 4A), which resulted in a substitution of the 336^th^ evolutionally conserved valine for leucine (p.Val336Leu) (Fig. 4B). This mutation has not been identified in the ExAC database. And *In silico* prediction (MutationTaster) reported a deleterious effect. The mutation was confirmed in all five living patients. Individual II-7 was detected with the mutation as well. Because the disorder presented with autosomal dominant inheritance in the current family, and the daughter of individual II-7 was obviously affected, we inferred that she was an asymptomatic mutation carrier. Furthermore, this mutation was not found in other unaffected family members or 300 unrelated healthy controls. Collectively, these results suggested that the p.Val336Leu mutation in the *PRKAG2* gene was potentially causative in the present family with cardiac hypertrophy and VPE. And the diagnosis of PS might be established in the affected members.

Notably, although the *PRKAG2* gene was included in our targeted sequencing panel, a previous genetic analysis of the index case did not identify the variation. We reevaluated the target region capture sequencing data using IGV after the mutation screening by whole-exome sequencing, and found that the exon 9 sequence, in which the mutation was located, was not covered in the previous sequencing.

### cDNA analysis

**Figure 5:**
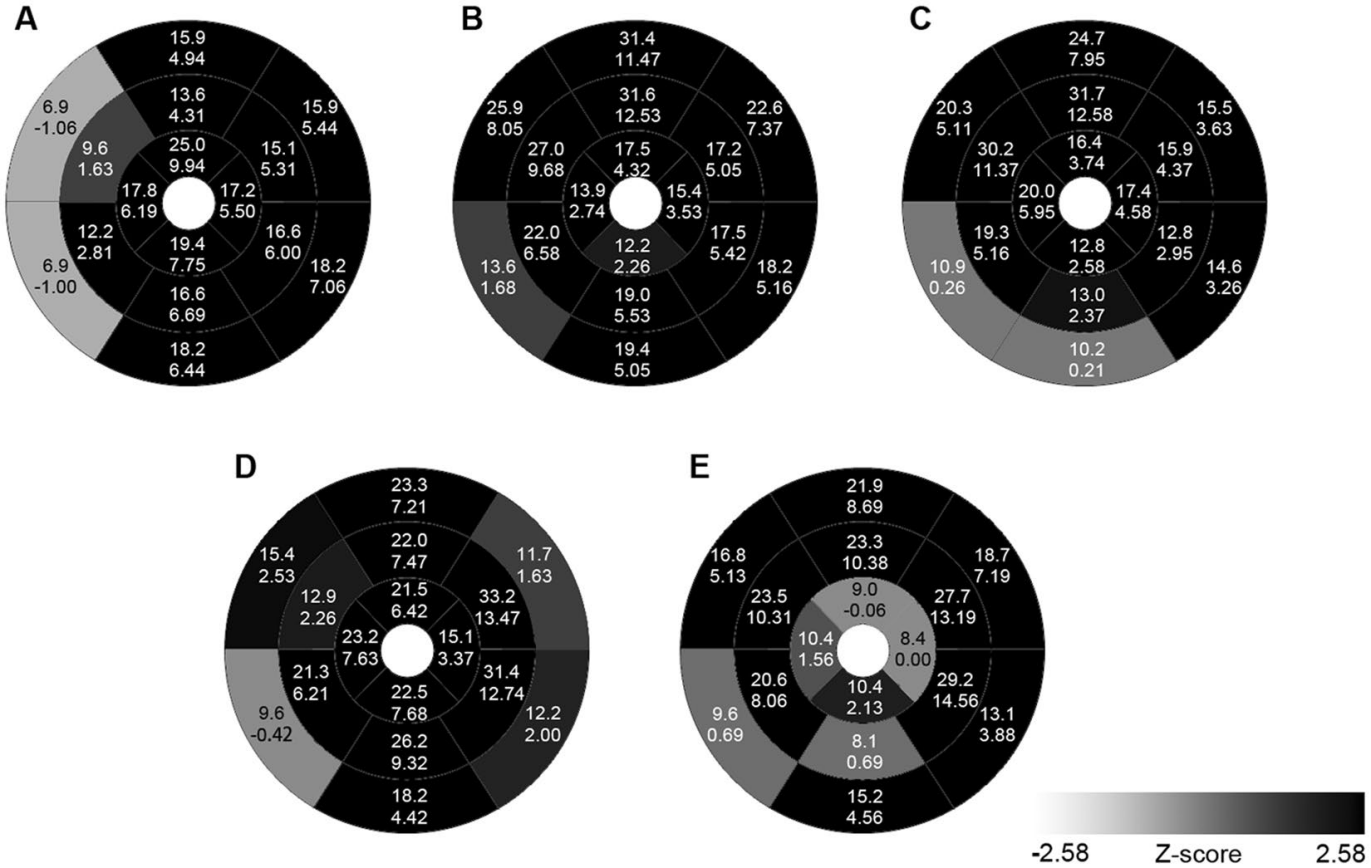
Distribution of left ventricular hypertrophy in patients with *PRKAG2* p.V336L mutations in the current family. A, II-3; B, II-5; C, II-9; D, III-5; and E, III-6. Upper and lower values indicate absolute (mm) and standardized (z-score) end-systolic maximal wall thickness in segments. Each segment is labeled with gray scaling according to z-score.

Because it is the first nucleotide of exon 9 in the *PRKAG2* gene that is altered, we evaluated the effect of this mutation on mRNA splicing. The sequence analysis of *PRKAG2* cDNA fragments from the proband containing a region between Exon 7–11 did not reveal any splicing defects (Fig. 5C).

### CMR characteristics

A CMR study was performed in the five affected family members (individuals II-3, II-5, II-9, III-5, and III-6). Detailed CMR characteristics are presented in Table 2. The proband was the only one who had preserved age, gender, and BSA standardized left ventricular end-diastolic volume (LVEDV), stoke volume (SV), and LVEF. The other four patients had much higher indexed LVEDV, and two patients (II-5 and III-6) had concurrently elevated indexed SV. Apparent impaired LVEF was observed in patient III-5, which decreased to 46% (z-score, −4.67). Notably, all studied patients had left ventricular (LV) mass above age and gender limits, and four patients (aged 49, 45, 24, and 28 years) had remarkably elevated LV mass (208.9 g/m^2^ [z-score, 15.87], 151.3 g/m^2^ [9.09], 233.4 g/m^2^ [18.75], 149.7 g/m^2^ [11.56]).

**Table 2:** CMR characteristics of five patients with PRKAG2 mutation in the current family.

Patient	II-3	II-5	II-9	III-5	III-6	z-score
Left ventricle						
Left atrial dimension, mm	31	46	36	21	28	
Left ventricular end-diastolic dimension, mm	46	55	55	41	48	
End-diastolic volume, ml	152.6	264.6	225.4	246.3	196	
End-diastolic volume index, ml/m^2^	84.0	134.3	115.2	134.6	116	4.7 (1.0, 6.1)
End-systolic volume, ml	43.9	99.6	98.3	132.9	66.9	
End-systolic volume index, ml/m^2^	24.2	50.5	50.2	72.7	39.5	4.2 (0.04, 8.3)
Stroke volume, ml	108.6	165.0	127.1	113.3	129	
Stroke volume index, ml/m^2^	59.8	83.7	65	62	76.3	2.0 (1.5, 5.0)
Cardiac output, l/min	6.7	10.6	9.53	6.01	6.21	
Cardiac output index, l/(min·m^2^)	3.7	5.4	4.9	3.3	3.7	
LVEF, %	71.2	62.4	56.4	46.0	66.0	−1.0 (−4.7, −0.9)
Mass, g	196.8	411.7	296.0	426.9	253.0	
Mass index, g/m^2^	108.4	208.9	151.3	233.4	149.7	11.6 (6.1, 18.8)
Left ventricle hypertrophy	+	+	+	+	+	
Maximal wall thickness, mm	25	31.6	31.7	33.2	29.2	12.5 (9.9, 14.6)
LGE presence	−	+	+	+	−	
Right ventricle						
Right ventricle hypertrophy	−	−	+	+	−	
LGE presence	−	−	−	+	−	

LVEF, left ventricular ejection fraction; LGE, late gadolinium enhancement.

All five patients had conspicuous LV hypertrophy in an concentric pattern (Fig. 5), with a median maximal wall thickness of 31.6 mm (range 25–33.2 mm) and z-score of 12.5 (range 9.9–14.6) (Table 2). Patient II-3 had a characteristic spade-like configuration of the cavity (see Supplementary Fig. S1), with a maximal wall thickness of 25 mm located in the apical anterior segment (Fig. 5A). Patients II-5 and II-9 had high-normal LVED dimensions, and hypertrophy predominantly in the mid-anterior and mid-anteroseptal segments (Fig. 5B and C). Patient III-5, who had the most markedly increased LV mass, had hypertrophy predominantly in the mid-lateral segments with a maximal wall thickness of 33.2 mm (z-score, 13.47) (Fig. 5D). The right ventricle was also involved with a maximal wall thickness of 13.9 mm located in the mid-anterior segment. Prominent trabeculae were observed in the mid-distal part of the lateral wall of patient III-6 (see Supplementary Fig. S1), whose maximal wall thickness was located in the mid-inferolateral segment (Fig. 5E).

LGE was present in three of the five patients (60%) examined by CMR (Fig. 3 and Supplementary Fig. S1). Hyperenhancement primarily focused on the mid-distal hypertrophic regions, and all three LGE positive patients presented with a subendocardial pattern. Transmural hyperenhancement within the apical regions was also revealed in patients II-5 and II-9, while ill-defined LGE throughout the LV myocardium was observed in patient III-5 (Fig. 3F–J).

## Discussion

In this study, we investigated the genetic defect and CMR characteristics in a three-generation Chinese family with cardiac hypertrophy and VPE. A novel missense mutation of the *PRKAG2* gene (c.1006 G > T, p.V336L) was identified by whole-exome sequencing. And the diagnosis of PS was made in the affected members. Myocardial morphology and function were evaluated form CMR findings of five affected members.

AMPK is an important energy-sensing enzyme that is activated by increases in the AMP/ATP ratio, and is deeply involved in cellular ATP metabolism^7^. AMPK exists as a heterotrimeric complex composed of a catabolic α subunit and regulatory β and γ subunits with multiple isoforms for each subunit^7^. Two isoforms of the γ subunit (γ1and γ2) are expressed in cardiac tissue, with predominance of γ2 subunit^8^. The AMPK-γ2 subunit, which is encoded by *PRKAG2*, contains four tandem repeats of sequence motifs, known as CBS repeats (CBS 1–4), which assemble to generate four potential nucleotide-binding sites for adenosine-containing ligands, such as AMP, ADP and ATP^18^. Activation of AMPK is triggered by AMP binding to the γ subunit, which causes the conformational change of α subunit, promotes phosphorylation, and inhibits Thr172 dephosphorylation within the α subunit kinase domain^19^. Most of the reported disease-causing mutations occur in the highly conserved CBS domain region, disturbing the normal interaction between AMPK-γ2 subunit and adenosine-containing ligands. The mutation identified in the present family is located adjacent to the CBS1 domain, and replace the highly conserved valine, which potentially affect the tridimensional structure of AMPK and result in enzyme activity modification^4^. A recently reported causative mutation lies in the region between CBS1 and CBS2 as well, suggesting this domain interval has a critical function^16^. Moreover, a disease-causing mutation in the same position (V336) as that of the present family was recently identified in a three-generation pedigree^20^. All four family members carrying that mutation (p.V336A) showed severe left ventricular hypertrophy, obvious electrophysiological abnormalities, and a progressive course. The conspicuous phenotype seen with the substitution of this amino acid in two families suggests that this residue play an important role.

The molecular mechanisms underlying PRKAG2 phenotype are not clearly evident. Inconsistent results are obtained from previous studies regarding to the effect of the PRKAG2 mutation on the regulation of AMPK activity. *In vitro*, some studies showed impaired binding between AMP and AMPK-γ2 subunit but increased basal AMPK activity^21, 22^, while other studies showed reduction in both AMP binding and AMPK activity^6, 23^. In mouse models, variable AMPK activity is observed. AMPK is constitutively activated in the N488I hearts^24^, while AMPK activity in the R302Q hearts is low^25^. The R531G hearts have normal AMPK activity at 1 week, and decrease in the activity is observed after glycogen accumulation^26^. Increased glycogen content is usually observed in the cardiac histopathological biopsies from patients with PS and model mice. In the TG^N488I^ mouse model, aberrantly activated AMPK may increase glucose uptake by accelerating translocation and expression of glucose transporters, and subsequently, persistent allosteric activation of glycogen synthase by abundant glucose-6-phosphate promotes glycogen storage^18^.

Electrocardiographic abnormalities are common in PS, particularly short PR interval, which is largely attributed to excessive glycogen accumulation in myocytes^4, 27^. During cardiogenesis, collagen-containing fibrous rings separate the atria and ventricle, and can be disrupted by glycogen-filled myocytes, resulting in VPE and reciprocating arrhythmias^24, 28^. Atrioventricular bypass tract, which was observed in the proband in the current PS family, is the most described accessory pathway in PS cases. Besides, nodoventricular and fasciculoventricular pathways are also observed in patients with PS, suggesting an important role for *PRKAG2* in development of the cardiac conduction system^29–31^.

It is reported that more than 50% of PS patients present with LVH^4^, yet excessive glycogen deposition may not totally account for the cardiac hypertrophy. A recent study showed that the contribution of glycogen to increased heart mass was <5% in TG^T400N^ mice which recapitulates symptoms of patients with *PRKAG2* T400N mutation^32^. Proliferation and hypertrophy pathways are found to be involved in the development of PRKAG2 cardiomyopathy. In the TG^T400N^ mouse model, Banerjee *et al*.^32^ demonstrated that early activation of the NF-κB and Akt signaling pathway, which are triggered by inappropriate AMPK activation, mediated cardiac hypertrophy. And recently, Kim and his colleagues suggested that the FoxO and mTOR signaling cascades were involved in activation of the proliferation and hypertrophy pathway related to AMPK-γ2 mutation^33^.

To date, 21 mutations have been reported to be associated with PS (Fig. 4B), and most of these are missense mutations, with the most frequently reported mutations being p.R302Q and p.N488I^4^. The clinical phenotypes are various among the *PRKAG2* mutation families. Some affected families only present with VPE and conduction system disease with the absence of cardiac hypertrophy^2, 34^, while in other affected families, cardiac hypertrophy is the primary feature^1, 3^. Even in the same family, affected members sharing with the same mutation can have different manifestations. Cardiac hypertrophy is a prominent feature in the current family, which is found in all affected members. A patient presented with biventricular hypertrophy, while only the left ventricle is involved in other affected family members. In comparison with cardiac hypertrophy, VPE is found in 4 members, and only two affected members have conduction system dysfunction. Mutations causing PS have a high penetrance which is estimated to be 99%^4^. Individual II-7 with the p.V336L mutation in the present family fails to be found with cardiac hypertrophy, VPE and any conduction system abnormalities, and she is inferred as an asymptomatic mutation carrier.

The role of CMR in the evaluation of metabolic cardiomyopathies has been emerged these years. To our knowledge, only nine patients with PS have been reported with CMR findings^11, 15–17^ (Table 3), and most of them are case reports. In this study, we provide additional information in CMR characteristics of PRKAG2 cardiomyopathy from five affected family members. We found that LV hypertrophy is diffuse (≥10 segments [≥62% of LV]) with a concentric pattern in five patients, although marked hypertrophy was observed in certain segments. Most recently, Yogasundaram *et al*.^17^ reported a 31 year-old male PS patient with concentric LVH, also demonstrated by CMR. Concentric hypertrophy is the most common pattern in metabolic and infiltrative disorders^35^. However, a recent CMR study showed an eccentric pattern in six patients with *PRKAG2* defects^16^. As two patients with remarkably increased LV mass presented with diffuse hypertrophy, hypertrophy in the interventricular septum was much more predominant. The other four patients exhibited a non-symmetric mid-infero-lateral pattern of hypertrophy. In two previous reports, asymmetric hypertrophy was confirmed by CMR in two PS patients^11, 15^. Existence of multiple morphological variants, as in the case of HCM^36^, is a possible explanation. In a cohort of 32 *PRKAG2* mutation carriers with LVH, an echocardiography study revealed different hypertrophy patterns, including concentric (59%), asymmetric (38%), and distal (3%) hypertrophy^3^. Interestingly, four patients examined by CMR in the current family exhibited increased end-diastolic and end-systolic volumes, with concentric hypertrophy. This may imply a progressive disease course, as impairment of LVEF following SCD occurred in one patient, while enlargement of LV was observed in other two patients.

**Table 3:** CMR features of patients with PRKAG2 syndrome.

Report	PRKAG2 mutation	Sex/Age (years)	Event	HCM pattern	LGE	LGE location
Fabris *et al*.^15^	R302Q	M/17	−	asymmetric (lateral)	NA	NA
Sternick *et al*.^11^	R302Q	M/18	MI	asymmetric (septal)	+	interventricular septum (subendocardial) + anterior (subendocardial)
Pöyhönen *et al*.^16^	R302Q	M/48	PMI	diffuse, eccentric	+	anteroseptal (patchy) + anterior (patchy)
Pöyhönen *et al*.^16^	R302Q	M/26	−	intermediate, asymmetric (mid-infero-lateral)	−	−
Pöyhönen *et al*.^16^	R302Q	M/24	–	focal (mid-infero-lateral)	−	−
Pöyhönen *et al*.^16^	R302Q	F/23	PMI	diffuse, eccentric	+	NA
Pöyhönen *et al*.^16^	R302Q	M/16	−	Asymmetric (mid-infero-lateral)	−	−
Pöyhönen *et al*.^16^	H344P	F/17	−	intermediate, asymmetric (mid-infero-lateral)	−	−
Yogasundaram *et al*.^17^	R302Q	M/31	PMI	diffuse, concentric; RV involvement	+	throughout the myocardium (patchy, midmyocardial) + apical (midmyocardial, subepicardial)
The current study	V336L	F/53	−	diffuse, concentric	−	−
The current study	V336L	M/49	LA and LV enlargement	diffuse, concentric	+	mid-cavity (global subendocardial and intramyocardium) + apical (global subendocardial and transmural)
The current study	V336L	M/45	LV enlargement	diffuse, concentric	+	mid-cavity (global subendocardial) + apical (global subendocardial and transmural)
The current study	V336L	M/24	SCD	diffuse, concentric; RV involvement	+	basal inferior/lateral (subendocardial) + mid-cavity (global subendocardial and intramyocardium) + apical (global subendocardial and transmural)
The current study	V336L	F/27	−	diffuse, concentric	−	−

M, male; F, female; LA, left atrium; LV, left ventricle; RV, right ventricle; NA, not available; MI, myocardium infarction; SCD, sudden cardiac death; PMI, pacemaker implantation.

One of the most prominent CMR characteristics of PRKAG2 cardiomyopathy in the present study was global subendocardial hyperenhancement in mid-distal hypertrophic regions, with locally transmural hyperenhancement in the apical region. Global transmural or subendocardial LGE is the most frequent observation in cardiac amyloidosis^37^. Nevertheless, the basal subendocardium was not involved in our patients, while in other reports, PS patients with non-global hyerenhancement were shown^11, 16, 17^. Extensive LGE in HCM is suggested to be an independent prognostic marker for SCD or appropriate implantable cardioverter-defibrillator discharges^38^. In the current study, systolic function was impaired and a SCD event occurred in one patient with biventricular involvement of LGE. The other two patients with positive LGE developed enlarged LV. Additionally, three PS patients with mild to extensive LGE were reported to undergo pacemaker implantation^16, 17^. Myocardial infarction also occurred in a PS patient with localized gadolinium uptake in the interventricular septum^11^. Therefore, we propose that LGE may serve as a risk marker for SCD and other adverse outcomes. However, because of the small size of PS patients with CMR examinations, further studies are needed to determine the clinical relevance between presence and extent of LGE and adverse outcome in patients with *PRKAG2* defects.

In summary, we report a novel *PRKAG2* mutation that is associated with PS in a Chinese family. Molecular screening for *PRKAG2* mutations should be considered in patients who exhibit cardiac hypertrophy coexisting with VPE. CMR offers advantages for better characterization of PRKAG2 cardiomyopathy in PS and is a valuable aid for diagnosis. PS cardiac hypertrophy may present with multiple morphologies, but with dominance of a concentric pattern. CMR may play a potential role in assessment of risk in patients with PS.

## Methods

### Ethics statements

This study was approved by the Ethics Committee of Fuwai Hospital. All participants provided written informed consent. The methods were performed in accordance with the approved guidelines.

### Clinical evaluation

A Chinese family with a HCM phenotype was identified and investigated. Family members were evaluated by collecting a detailed medical history, physical examination, 12-lead electrocardiogram (ECG), 24-hour ambulatory ECG, and transthoracic echocardiography. When appropriate, CMR was conducted in patients with cardiac hypertrophy. Cardiac hypertrophy was defined by echocardiography or CMR as increased LV wall thickness ≥15 mm in one or more myocardial segments^35^. VPE was defined by a short PR interval (<120 ms)^20^. Presence of a short PR interval, widened ORS interval (>120 ms) and an abnormal QRS vector (delta-wave) was classified as Wolff-Parkinson-White (WPW) syndrome^20^.

CMR images were transferred to a workstation (Siemens Medical Systems) for analysis. In each patient, a 17 segment model was made from three short axis sections, according to American Heart Association criteria^39^. Left atrial and LV end-diastolic diameter, LV volumes, LVEF, LV mass, and maximal thickness of hypertrophied segments were measured. All parameters were standardized to age, gender, body surface area (BSA) and normal references (z-scores)^40, 41^. Late gadolinium enhancement (LGE) was qualitatively determined for each myocardial segment by reviewing all short and long axis contrast-enhanced images. LGE patterns were classified as subendocardial, subepicardial, mid-myocardial, or transmural (≥75% of any segmental wall thickness) by visual analysis^42^.

### Whole-exome sequencing

Genomic DNA was extracted from peripheral blood leukocytes using the QIAamp DNA Blood Midi Kit (QIAGEN, Hilden, Germany), according to standard protocols. The proband had previously been screened using a targeted sequencing panel containing 64 candidate genes reported to be causative of inherited cardiomyopathy^43^. No pathogenic mutations were detected. Next, three affected individuals (II-9, III-6, and III-8) and one unaffected individual (II-2) were selected for whole-exome sequencing. The Agilent SureSelect Human All Exon V5 capture kit (Agilent, Santa Clara, CA, USA) was used for exome capture. Samples from the family quartette were multiplexed on a single lane and 101-bp, paired-end sequencing was performed using Illumina’s HiSeq4000 platform (Illumina, Inc, San Diego, CA, USA) to an average depth of 141×.

Sequence data were aligned to the human reference genome (GRCh37/hg19) with BWA followed by sorting and marking of duplicate reads using Picard (version 2.4, http://broadinstitute.github.io/picard/). Local realignment of insertions/deletions (indels) and base quality score recalibration were performed using GATK (version 3.6, https://www.broadinstitute.org/gatk/). GATK was also used to call and filter variants within a genome–wide region. The resultant variants were annotated with ANNOWAR^44^ and sequentially filtered using the following criteria: (1) variants with an East Asian minor allele frequency ≥0.01 in the databases for 1000 Genomes Project (http://browser.1000genomes.org) and Exome Aggregation Consortium (ExAC, http://exac.broadinstitute.org) were removed; (2) variants in exons and splicing sites were retained; (3) certain types of variants were retained, including nonsynonymous, frameshift, nonframeshift insertion/deletion, stopgain and unknown; (4) variants in genes expressed in the heart according to the Human Protein Atlas (www.proteinatlas.org)^45^; and (5) variants that passed manual confirmation using the Integrative Genomics Viewer (IGV, http://software.broadinstitute.org/software/igv)^46^ were retained. Effects of variants on splicing signals were evaluated using the Human Splicing Finder (version 3.0, www.umd.be/HSF3/HSF.html)^47^.

Candidate variants were validated by Sanger sequencing. Three hundred unrelated healthy control samples were subjected to variant examination.

### Reverse transcription-polymerase chain reaction (RT-PCR)

Total RNA was isolated from peripheral blood leukocytes collected from individuals I-2 and II-3 using TRIzol LS reagent (Life Technologies, Grand Island, NY, USA). Reverse transcription reaction was performed using the GoScript Reverse Transcription System (Promega, Madison, WI, USA), according to the manufacturer’s protocol. cDNA sequence containing the mutation site was amplified by PCR using a forward primer located in exon 7 of the *PRKAG2* gene (5′- TCGTTCCAACCAGTTCAAAGC-3′), and a reverse primer located in exon 11 (5′-GTTCTGCTTCATGAAGGCAGG-3′). The resulting PCR products were analyzed by Sanger sequencing.

### Data Availability

All data generated or analysed during this study are included in this published article (and its Supplementary Information files).

## Electronic supplementary material

Supporting Information
