# Supplementary material for: A novel PRKAG2 mutation in a Chinese family with cardiac hypertrophy and ventricular pre-excitation
Source: Sci Rep. 2017 May 25;7:2407. doi: 10.1038/s41598-017-02455-z (PMC5445094; doi:10.1038/s41598-017-02455-z)
Supplement: Supplementary file 1 — Supporting Information [file 41598_2017_2455_MOESM1_ESM.pdf]

Supplementary Information

A novel *PRKAG2* mutation in a Chinese family with cardiac hypertrophy and ventricular pre-excitation

Kun-Qi Yang[1¶], Chao-Xia Lu[2¶], Ying Zhang[1¶], Yan-Kun Yang[1], Jia-Cheng Li[2], Tian Lan[3], Xu Meng[1], Peng Fan[1], Tao Tian[1], Lin-Ping Wang[1], Ya-Xin Liu[1], Xue Zhang[2*], Xian-Liang Zhou[1*]

[1]Department of Cardiology, Fuwai Hospital, National Center for Cardiovascular Diseases, Chinese Academy of Medical Sciences and Peking Union Medical College, Beijing, China

[2]McKusick-Zhang Center for Genetic Medicine, State Key Laboratory of Medical Molecular Biology, Institute of Basic Medical Sciences, Chinese Academy of Medical Sciences and Peking Union Medical College, Beijing, China

[3]Department of Magnetic Resonance Imaging, Cardiovascular Imaging and Intervention Center, Fuwai Hospital, National Center for Cardiovascular Diseases, Chinese Academy of Medical Sciences and Peking Union Medical College, Beijing, China

Supplementary Figure and legend

Supplementary Figure S1

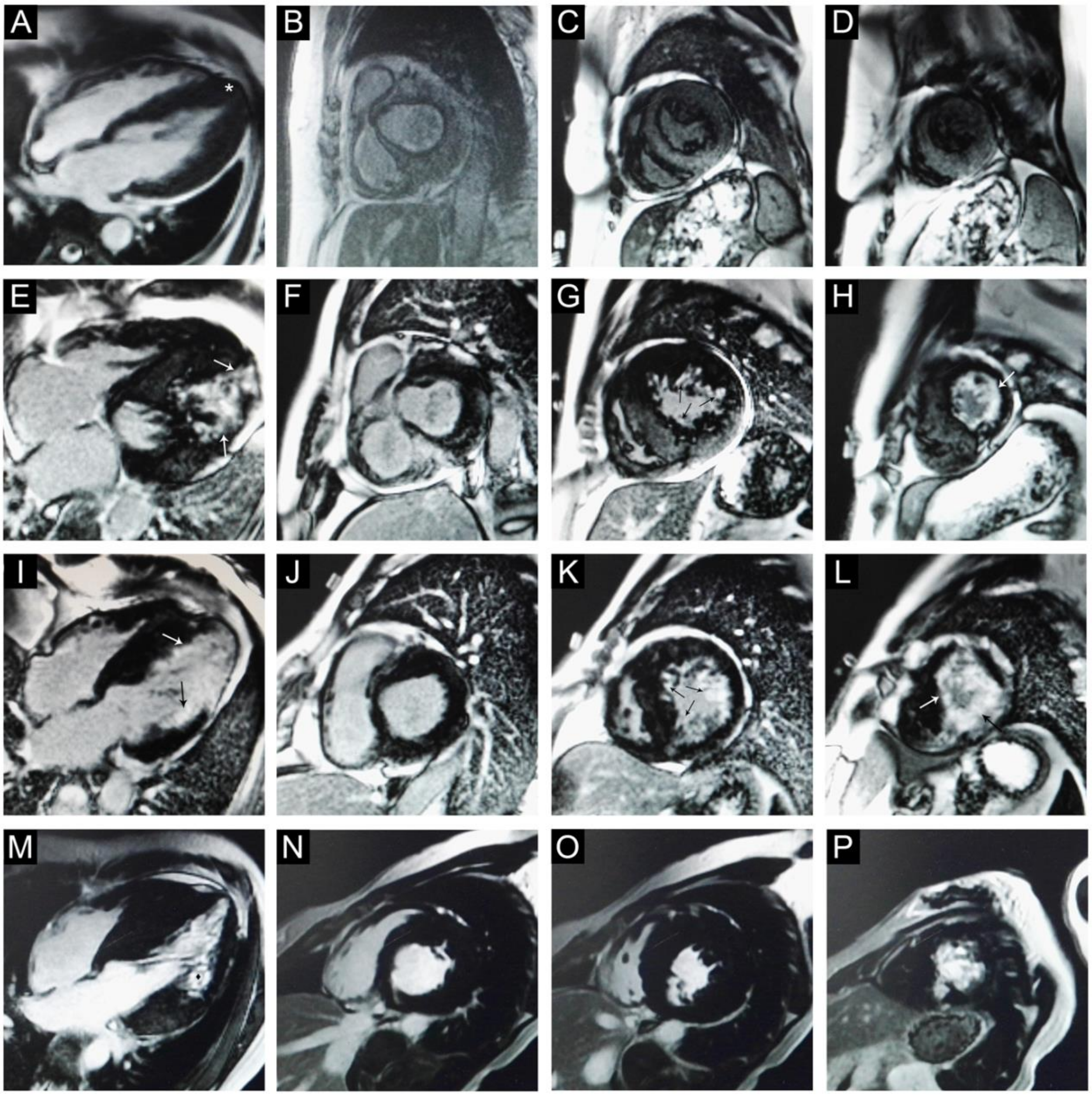

Supplementary Figure S1

Contrast-enhanced CMR images in four affected members with *PRKAG2* p.V336L mutations in the current family. A, II-3; B, II-5; C, II-9; and D, III-6. * indicates apical hypertrophy, arrows indicate late gadolinium enhancement, ♦ indicates noncompaction of ventricular myocardium.
